# Supplementary material for: A fault-intrusion-tolerant system and deadline-aware algorithm for scheduling scientific workflow in the cloud
Source: PeerJ Comput Sci. 2021 Nov 2;7:e747. doi: 10.7717/peerj-cs.747 (PMC8576554; doi:10.7717/peerj-cs.747)
Supplement: Supplemental Information 6 [file peerj-cs-07-747-s006.zip › Myproject1.1/docs/org/cloudbus/cloudsim/core/CloudSimShutdown.html]

CloudSimShutdown


JavaScript is disabled on your browser.


- Overview
- Package
- Class
- Use
- Tree
- Deprecated
- Index
- Help

- Prev Class
- Next Class

- Frames
- No Frames

- All Classes

- Summary:
- Nested |
- Field |
- Constr |
- Method

- Detail:
- Field |
- Constr |
- Method


org.cloudbus.cloudsim.core

## Class CloudSimShutdown

- java.lang.Object
- - org.cloudbus.cloudsim.core.SimEntity
  - - org.cloudbus.cloudsim.core.CloudSimShutdown

- All Implemented Interfaces:
  :   java.lang.Cloneable

  ---

    

  ```
  public class CloudSimShutdown
  extends SimEntity
  ```

  CloudimShutdown waits for termination of all CloudSim user entities to determine the end of
  simulation. This class will be created by CloudSim upon initialisation of the simulation, i.e.
  done via CloudSim.init() method. Hence, do not need to worry about creating an object of
  this class. This object signals the end of simulation to CloudInformationService (GIS) entity.

  Since:
  :   CloudSim Toolkit 1.0

- - ### Field Summary

    - ### Fields inherited from class org.cloudbus.cloudsim.core.SimEntity

      `FINISHED, HOLDING, RUNNABLE, WAITING`
  - ### Constructor Summary

    Constructors

    Constructor and Description

    `CloudSimShutdown(java.lang.String name,
    int numUser)`

    Allocates a new CloudSimShutdown object.
  - ### Method Summary

    Methods

    | Modifier and Type | Method and Description |
    | --- | --- |
    | `void` | `processEvent(SimEvent ev)` The main method that shuts down hostList and Cloud Information Service (GIS). |
    | `void` | `shutdownEntity()` This method is invoked by the `Simulation` before the simulation finishes. |
    | `void` | `startEntity()` This method is invoked by the `Simulation` class when the simulation is started. |

    - ### Methods inherited from class org.cloudbus.cloudsim.core.SimEntity

      `cancelEvent, clone, getEventBuffer, getId, getName, getNextEvent, getNextEvent, getState, numEventsWaiting, numEventsWaiting, pause, run, schedule, schedule, schedule, schedule, scheduleFirst, scheduleFirst, scheduleFirst, scheduleFirst, scheduleFirstNow, scheduleFirstNow, scheduleFirstNow, scheduleFirstNow, scheduleNow, scheduleNow, scheduleNow, scheduleNow, selectEvent, send, send, send, send, sendNow, sendNow, sendNow, sendNow, setEventBuffer, setId, setState, waitForEvent`
    - ### Methods inherited from class java.lang.Object

      `equals, finalize, getClass, hashCode, notify, notifyAll, toString, wait, wait, wait`

- - ### Constructor Detail


    - #### CloudSimShutdown

      ```
      public CloudSimShutdown(java.lang.String name,
                      int numUser)
                       throws java.lang.Exception
      ```

      Allocates a new CloudSimShutdown object.

      The total number of grid user entity plays an important role to determine whether all
      hostList should be shut down or not. If one or more users are still not finish, then the
      hostList will not be shut down. Therefore, it is important to give a correct number of total
      grid user entity. Otherwise, CloudSim program will hang or encounter a weird behaviour.

      Parameters:
      :   `name` - the name to be associated with this entity (as required by SimEntity class)
      :   `numUser` - total number of grid user entity

      Throws:
      :   `java.lang.Exception` - This happens when creating this entity before initialising CloudSim package
          or this entity name is null or empty

      See Also:
      :   `gridsim.CloudSim#init(int, Calendar, boolean)`
  - ### Method Detail


    - #### processEvent

      ```
      public void processEvent(SimEvent ev)
      ```

      The main method that shuts down hostList and Cloud Information Service (GIS). In addition,
      this method writes down a report at the end of a simulation based on
      reportWriterName defined in the Constructor.   
      **NOTE:** This method shuts down grid hostList and GIS entities either AFTER all
      grid users have been shut down or an entity requires an abrupt end of the whole simulation.
      In the first case, the number of grid users given in the Constructor must be
      correct. Otherwise, CloudSim package hangs forever or it does not terminate properly.

      **Specified by:**
      :   `processEvent` in class `SimEntity`

      Parameters:
      :   `ev` - the ev


    - #### startEntity

      ```
      public void startEntity()
      ```

      **Description copied from class: `SimEntity`**

      This method is invoked by the `Simulation` class when the simulation is started. This
      method should be responsible for starting the entity up.

      **Specified by:**
      :   `startEntity` in class `SimEntity`


    - #### shutdownEntity

      ```
      public void shutdownEntity()
      ```

      **Description copied from class: `SimEntity`**

      This method is invoked by the `Simulation` before the simulation finishes. If you want
      to save data in log files this is the method in which the corresponding code would be placed.

      **Specified by:**
      :   `shutdownEntity` in class `SimEntity`


- Overview
- Package
- Class
- Use
- Tree
- Deprecated
- Index
- Help

- Prev Class
- Next Class

- Frames
- No Frames

- All Classes

- Summary:
- Nested |
- Field |
- Constr |
- Method

- Detail:
- Field |
- Constr |
- Method
